# Alendronate Release from UHMWPE-Based Biomaterials in Relation to Particle Size of the GUR Powder for Manufacturing

**DOI:** 10.3390/ma12111832

**Published:** 2019-06-06

**Authors:** Michael Seidenstuecker, Julia Weber, Sergio H. Latorre, Brigitte Straub, Ulrich Matthes, Christian Friedrich, Hermann O. Mayr, Anke Bernstein

**Affiliations:** 1 G.E.R.N. Tissue Replacement, Regeneration & Neogenesis, Department of Orthopedics and Trauma Surgery, Medical Center-Albert-Ludwigs-University of Freiburg, Faculty of Medicine, Albert-Ludwigs-University of Freiburg, Hugstetter Str. 55, 79106 Freiburg, Germany; juli-weber@hotmail.de (J.W.); sergio.latorre@uniklinik-freiburg.de (S.H.L.); hermann.mayr@uniklinik-freiburg.de (H.O.M.); anke.bernstein@uniklinik-freiburg.de (A.B.); 2 Faculty Mechanical and Medical Engineering, Furtwangen University, Robert-Gerwig-Platz 1, 78120 Furtwangen, Germany; sbr@hs-furtwangen.de; 3 Freiburg Materials Research Center (FMF) and Institute for Macromolecular Chemistry of the Albert-Ludwigs University Freiburg, Stefan-Meier-Str. 21, D-79104 Freiburg, Germany; ulrich.matthes@fmf.uni-freiburg.de (U.M.); christian.friedrich@fmf.uni-freiburg.de (C.F.)

**Keywords:** UHMWPE, GUR1020, GUR1050, alendronate, osteoporosis, aseptic loosening, drug release, HPLC

## Abstract

Ultra-high molecular weight polyethylene (UHMWPE) is widely used in endoprosthetics and has been the subject of countless studies. This project investigates the dependence of alendronate (AL) release on the molecular weight of the UHMWPE used (GUR1020 and GUR1050). A 0.5 wt% AL was added to the UHMWPE during the production of the moldings. In addition to the 14-day release tests, biocompatibility tests such as live dead assay, cell proliferation assay (WST) and Lactate dehydrogenase test (LDH) with MG-63 cells as well as a tensile test according to DIN EN ISO 527 were carried out. The released AL concentration was determined by HPLC. A continuous release of the AL was observed over the entire period of 2 weeks. In addition, a correlation between molar mass and AL release was demonstrated. The GUR1020 showed a release four times higher than the GUR1050. Both materials have no negative influence on the proliferation of MG-63 cells. This was also confirmed in the live/dead assay by the increase in cell count. No cytotoxicity was detected in the LDH test. The addition of 0.5 wt% AL increased the elongation at break for GUR1020 by 23% and for GUR1050 by 49%. It was demonstrated that the choice of UHMWPE has an influence on the release of AL. The particle size in particular has a strong influence on the release behavior.

## 1. Introduction

**Figure 1:**
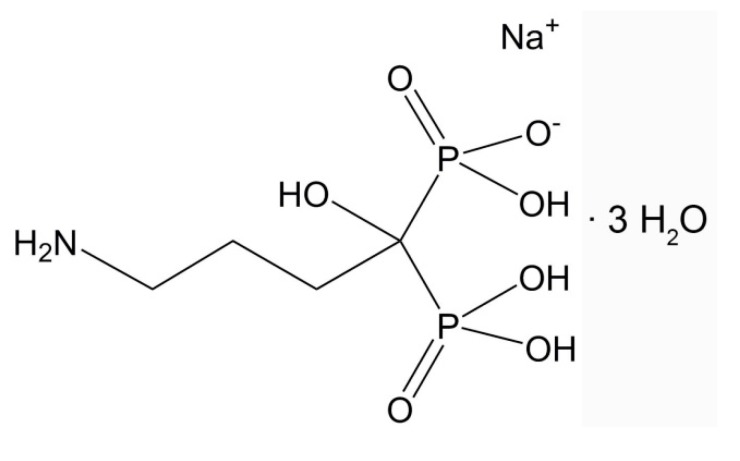
Alendronate sodium.

The fact that we are living so much longer means that the percentage of people eventually needing an artificial joint will also rise dramatically. In 2016, there were 187,319 initial implantations of total knee replacements (TKRs), and 24,940 revision surgeries for TKRs in Germany [1]. The annual operative frequency of initial endoprosthetic hip and knee surgery among Germans has remained stable and not risen since 2007. Initial hip interventions in the over-70 age group amounted to 1.1% (in 2007 and 2014), and initial knee interventions 0.7% (2007) and 0.6% (2014). The operative frequency in the entire German population was 0.26% in 2014 (hips) and 0.19% (knees) [2]. However, 6.5% of the TKR had to be replaced within the first two postoperative years. Of the revised interventions, 55% were due to aseptic loosening [3], a process caused by minute polyethylene particles abraded from the inlay made of ultra-high molecular weight polyethylene (UHMWPE) in knee endoprosthesis [4]. Despite good sliding characteristics, wear particles arise that are phagocytized by macrophages. In reacting to these non-decomposable particles, the macrophages discharge inflammatory mediators that increase osteoclastic activity [5]. The central mechanism of abrasion particle-induced osteolysis is elevated bone resorption through osteoclasts. The therapeutic targeting of osteoclast function is a logical means of treating or at least minimizing the occurrence of aseptic loosening after total joint replacement. The therapy for an endoprosthesis loosened by periprosthetic osteolysis usually entails removal of the old and implantation of a new endoprosthesis. Such surgical revisions are associated with a high degree of morbidity and impaired function. More recent approaches to osteolysis suppression focus on understanding and manipulating osteolysis at the molecular level through pharmacological intervention [6]. Potential biological treatments involve reagents such as bisphosphonate, statins, and antagonists for the Receptor Activator of Nuclear Factor Kappa B Ligand (RANKL), considered a signal transducer of osteoclasts onto osteoblasts. Various studies have demonstrated that nitrogenous bisphosphonates such as zolendronate can inhibit abrasion-induced osteolysis and lead to increased peri-implant bone density [7,8]. Alendronate belongs to the group of bisphosphonates and is already being applied in medical therapy and prophylactically for osteoporosis [9]. Bone structure is made denser by the consistent delivery of alendronate, making the bone less fracture prone [10,11]. These effects rely on inhibiting osteoclastic activity. Figure 1 illustrates the chemical structure of alendronate sodium (C_4_H_12_NNaO_7_P_2_ ∙ 3 H_2_O).

The type of polyethylene (PE) usually employed to manufacture prostheses is UHMWPE [12]. It triggers fewer abrasion particles than PE with a lower molar mass, as well as greater stability and better gliding qualities. In working on this project, the focus was on the polyethylenes GUR1020 and GUR1050 [13]. The materials used in our experiments (GUR1020 und GUR1050) are medical substances and categorized as UHMWPE. These materials have been administered around the world for orthopedic purposes (e.g., joint replacements). Their only differences lie in their molecular weight [13]: GUR1020 (3.5 ∙ 10^6^ g/mol) and GUR1050 (5.5–6 ∙ 10^6^ g/mol).

AL was integrated into UHMWPE as an active ingredient. As such, the active ingredient’s systemic concentration must be kept as low as possible to prevent potential side effects, something only possible when it is applied locally onto the target cells. The active ingredient AL must be integrated homogeneously within the polymer matrix so that the concentration can be achieved that is necessary to prevent the onset of particle-induced periprosthetic osteolysis prior to OA. This concentration is maintained during the endoprosthesis’ lifetime by the particles released during the abrasion process and the associated release of the active substance. 

In the release experiments described in the literature of alendronate from UHMWPE [14,15,16], abrasion particles were produced from which AL was later released [14,16], and AL release attempts were made from GUR1020 [15]. However, no attempt has been made until now to investigate the relationship between release behavior and the particle size. In this study, the influence of the particle size of different UHMWPEs on the release behavior of AL was investigated. The biocompatibility of GUR1020/1050 and alendronate composites was also evaluated, so that alendronate can be eventually added to the prosthesis material during the manufacturing process.

## 2. Materials and Methods 

### 2.1. Reagents and Materials

GUR1020 und GUR1050 were obtained from Ticona/Celanese (Dallas, USA). Alendronate (AL) sodium, o-Phtadialdehyde (OPA), 2-Mercaptoethanol (2ME) and Tetrabutylammoniumperchlorate (TBA) were purchased from Sigma Aldrich (Darmstadt, Germany). Sodium hydrogen phosphate, di-sodium hydrogen phosphate and Acetonitril (ACN) were from Carl Roth (Karlsruhe, Germany). All these chemicals met HLPC standards. 

### 2.2. Sample Preparation

**Figure 2:**
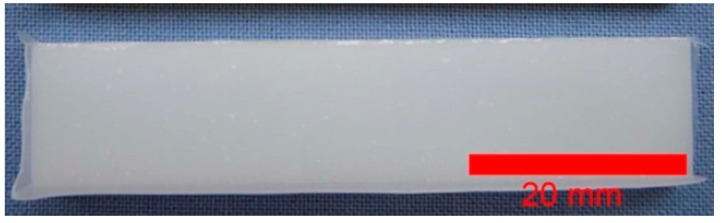
GUR molds after pressing, red bar = 20 mm.

Sample preparation was similar to Qu et al. [16], but with a different temperature control to keep the AL from disintegrating. A GUR-alendronate batch containing 0.5 wt% alendronate was prepared. To do this, the AL was dissolved in 1:5 ethanol:double-distilled water and then mixed with GUR-powder (1020 or 1050). The resultant dispersion was then dried at 35 °C in a vacuum drying oven (Memmert, Schwabach, Germany). A total of 1.55 g of each sample was pressed into shape (60 × 13 × 2 mm) at 140 °C with 60 bar, with a slab press type P 200 P with vacuum function (Dr. Collin GmbH, Ebersberg, Germany) (see Figure 2). There were also samples from pure GUR (1020/1050) without adding any chemical agents produced. 

The resultant molds (cf. Figure 2) were cut into sections measuring 10 × 13 × 2 mm for the release experiments. For the cell experiments, cylinders measuring ø 10 mm were die cut out of the molds. These round test specimens were better suited for cell-culture slides, whereas the angled ones are more appropriate for release experiments thanks to having a larger surface. 

### 2.3. Characterization of the GUR and the Molds

#### 2.3.1. Particle Size

The particle size was determined using a Morphologi G3 (Malvern Panalytical, Malvern, UK) particle measuring device. A defined volume was introduced into the atomizer of the Morphologi G3 with a spatula and atomized at 2 bar for 30 ms on the slide. Then at least 3000 particles per GUR with 5× magnification were recorded and measured. The measurements were repeated 3 times.

#### 2.3.2. Weight and Dimensions

After sawing or punching out the GUR bodies, the exact width and thickness were determined using an electronic sliding calliper (Burg-Wächter, Wetter, Germany) and the weight of at least 10 samples per GUR/composite was determined using a Sartorius precision balance.

#### 2.3.3. Surface Roughness

A total of 6 samples from each batch (GUR1020, GUR1050, GUR1020AL, GUR1050AL) were examined under a 3D laser-scanning microscope (3DLSM) (VK-X210, Keyence, Osaka, Japan), at room temperature and at 1000× magnification, concentrating on their surface structure. A surface measuring 64,000 µm² was examined to assess each sample’s surface roughness, and multiple measurements were taken at five different positions on each sample. The arithmetical mean height S_a_ of each sample was determined corresponding to JIS B0601:2001 (ISO 4287:1997).

#### 2.3.4. Tensile Testing 

The tensile tests were performed on a universal testing machine Z005 (Zwick/Roell, Ulm, Germany) according to DIN EN ISO 527 with a 5-kN load cell. The specimens were produced as described previously and die cut in Form 5A for the tensile tests. Following the DIN norm, first the young´s module at 1 mm/min velocity and then the tensile strength until total failure at a velocity of 50 mm/min was tested. A total of 6 samples of each GUR and composite specimen were assessed. 

#### 2.3.5. Crystallinity

The crystallinity of specimens with and without AL was assessed via differential scanning calorimetry (DSC). These DSC measurements were taken on a DSC 204F1 Phoenix (Netzsch Gerätebau GmbH, Selb, Germany) within a 20–200 °C temperature range with a heating-up rate or cooling-down rate of 10 K/min under a nitrogen atmosphere. Four samples of each GUR and composite were analyzed.

The percent crystallinity was measured in dry N2 according to ASTM D3418-03. The enthalpy of fusion (H_f_) was calculated by integrating the DSC endotherm from the second heating curve. According to the UHMWPE Biomaterials Handbook [17], the H_f_ of 100% crystalline UHMWPE equaled 288 J/g. The percent crystallinity was calculated by dividing the H_f_ of the sample by 288 J/g and multiplying by 100.

### 2.4. Release Experiments

Each mold for the release experiments (N = 10) was immersed in 2 mL twice-distilled water and weighed down with glass rings (outer diameter = 8 mm, inner diameter = 6 mm) to prevent them from floating to the surface. The release experiments were carried out over 14 days at 37 °C. The specimens were brought into motion with a shaking device (Rocker 2D basic, IKA, Staufen, Germany). The fluids were removed entirely at specific timepoints (1, 2, 3, 6, 8 und 14 days) and replaced with fresh double-distilled water (arium pro, Sartorius). These fluid specimens were then deep-frozen at −20 °C for later experimental use. The release behavior of 10 samples from each specimen was examined in triplicate. 

### 2.5. HPLC

#### 2.5.1. Equipment

HPLC analysis took place with a HPLC System (Shimadzu, Kyoto, Japan) consisting of 2 Nexera XR LC-20AD pumps and a SIC-30AC autosampler, CTO 20 AC column oven, DGU-20A5R Degasser, SPD-M20A PDA detector, RF 20A fluorescence detector and a CBM-20A controller. A Hamilton PRP-1 column (5 µm, 150 mm × 4.1 mm) was used to take HPLC measurements. 

#### 2.5.2. Buffer Preparation

The phosphate buffer pH 9.6 was produced after Sorensen [18]. The 3 wt% TBA was added; the pH value was tested and, if necessary, readjusted with phosphoric acid to pH 9.6. The buffer solution was filtered through a polycarbonate membrane with a pore size of 0.4 µm under a partial vacuum at 500 mbar prior to being employed at the HPLC.

#### 2.5.3. Derivatization Solution

A working solution according to Al Deeb et al. [19] was used. Therefore, 50 mg OPA was dissolved in 5 mL 0.05 M NaOH, then 250 µL 2ME was added and the solution was topped up with 0.05 M NaOH to 50 mL. A fresh batch of this solution was made every day. 

#### 2.5.4. Chromatographic Settings

The chromatographic separations and the subsequent quantifications were performed at room temperature using a reversed-phase HPLC column. The chromatogram was documented using the fluorescence detector (excitation: 333 nm, emission 455 nm). An isocratic solvent system consisting of a mixture of ACN/phosphate buffer pH 9.6 (15:85) with 3 wt% TBA according to Al Deeb et al [19] at a flow rate of 1 mL/min was used.

#### 2.5.5. Calibration Curve

A stock solution containing 1 mg/mL alendronate in 0.05 M NaOH was prepared. Aliquots were extracted from the stock solution for calibration, then transferred to HPLC vials, mixed 60 µL OPA/2ME into each one, and filled up to 1 mL with 0.05 M NaOH. After allowing 60 min reaction time, the specimens were analyzed via HPLC. The various concentrations (10–200 µg/mL) were produced fresh daily for the calibration.

#### 2.5.6. Preparing the Specimens

The samples obtained from the release experiments were mixed with 60 µL OPA/2ME and filled up to 1 mL, analogous to 2.4.5. These samples were then analyzed 60 min later via HPLC and a fluorescence detector (333 nm excitation, 455 nm emission).

### 2.6. Biocompatibility Investigations

The cell-culture experiments were performed with an MG-63 cell line (ATCC CRL-1427). To do this, the die-cut GUR specimens were first sterilized in an autoclave (Varioklav 135T) (HP Medizintechnik GmbH, Oberschleißheim, Germany) and then fixed onto the base of a 24-well plate with Futar D (Kettenbach GmbH & Co. KG, Eschenburg, Germany), a dental adhesive based on vinylpolysiloxane. A total of 5000 MG-63 cells were seeded onto each specimen and completely covered with medium, and then incubated in an incubator (Galaxy 170R, New Brunswick, Canada) for a specific time period. All biocompatibility tests involved at least three specimen GURs or composites at each time point, and each test was repeated three times. The specimens were examined microscopically under the BX51fluorescence microscope (Olympus, Tokyo, Japan) and BX51 light microscope (Olympus, Tokyo, Japan) with Software Stream Motion. In all the following investigations, at least three samples of each GUR or composite specimen was tested at each timepoint, and at least 5 different positions on each of those samples were examined microscopically at 5× and 10×magnification. The internal light source of the BX 51 was used for light microscopy and an external light source with λ = 490 nm was used for fluorescence microscopy. The filter we used enabled us to observe red and green fluorescing cells simultaneously. The cell count was performed by hand using the Photoshop CS6 counting software (Adobe, San José, USA). The mean values of cell count were calculated and then converted into cell count values per mm². 

#### 2.6.1. Live/Dead Assay

This staining procedure was performed after 24 h, 48 h and 72 h with “Live/Dead Cell Straining Kit II” from PromoKine. Calcein stains live cells green (Ex/Em 495 nm/ 515 nm), while EthD-III stains the dead ones red (Ex/Em 530 nm/635 nm). Microscopy was performed as stated previously. 

#### 2.6.2. Cell Proliferation Test (WST) 

For this test “Cell Proliferation Reagent WST-1” from Roche was used, measuring after 1, 2, 3, 7 and 10 days at λ = 450 nm and λ = 600 nm as a reference in a photometer (SPECTROstar^Nano^, BMG Labtech GmbH). The WST reagent was added at a ratio of 1:10 to the DMEM-F12 medium (without red phenol) and incubated for 2 h at 37 °C, 5% CO_2_. From each well, 100 µL of the liquid was then pipetted into a new 96-well plate in triplicate.

#### 2.6.3. LDH Test

A total of 25000 cells was seeded onto each specimen. This experiment was conducted with Roches “Cytotoxicity Detection Kit (LDH)”. Measurements were taken after 1, 2 and 3 days at λ = 490 nm in a photometer. In so doing, we added 100 µL freshly prepared LDH solution to each 100 µL of the medium used (phenol red free, 1% Pen Strep, 1% FBS Superior). These mixtures then had to undergo incubation for at least 30 min in the dark and were then measured photometrically.

### 2.7. Statistical Analysis

Data were expressed as mean ± standard deviation of the mean and analyzed by one-way analysis of variance (ANOVA). The level of statistical significance was set at p < 0.05. For statistical calculations, Origin 2018 Professional SR1 (OriginLab, Northampton, USA) was used.

## 3. Results

### 3.1. GUR and Specimen Characterization

#### 3.1.1. Particle Size

Particle size measurements showed different size distributions for the different GUR types. GUR1050 showed a larger proportion of small particles between 1 and 10 µm with 57%, whereas GUR1020 with 36.5% had a significantly smaller value for this particle size class. Looking at the small particle sizes of 1–30 µm together, the GUR1050 with 83.2% had a significantly higher proportion of small particles than the GUR1020 with 69.1%. Table 1 shows the comparison of the different particle size classes for the two GUR types. The mean particle diameter for GUR1020 was 54.3 ± 52.5 µm and 36.9 ± 38.3 µm for GUR1050. 

#### 3.1.2. Weights and Dimensions

The specimens were on average 9.51 ± 0.05 mm in width, 12.80 ± 0.15 mm in length, 2.09 ± 0.06 mm in height and weighed 0.24 ± 0.01 g. Table 2 shows the dimensions and weights of the specimens we used in our release experiments. Specimens GUR1020AL and GUR1050AL contained 0.5 wt% AL.

#### 3.1.3. Surface Roughness

**Figure 3:**
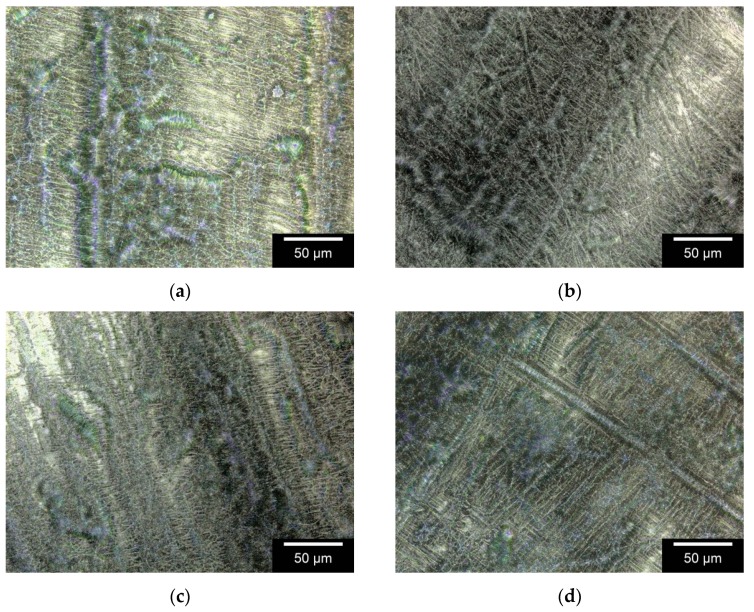
Surface roughness of specimens photographed with a Keyence 3DLSM VK-X210; 1000× magnification: **A**: GUR1020; **B**: GUR1050; **C**: GUR1020AL; **D**: GUR1050AL.

Figure 3 shows examples of various specimens photographed via 3DLSM at 1000× magnification. The images display a surface measuring 64,000 µm². 

A significant difference (p < 0.05) in the arithmetical mean height S_a_ between GUR1020 and GUR1020AL has been observed (cf. Table 3). The S_a_ of GUR1050 revealed no significant difference to that of GUR1050AL and neither did GUR1020 and GUR1050 significantly differ in their S_a_.

#### 3.1.4. Tensile Tests

**Figure 4:**
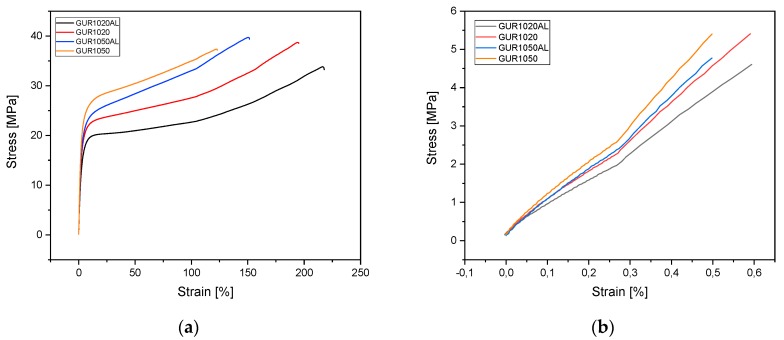
Stress–strain curves (**a**) GUR specimens; (**b**) the initial differences are magnified to facilitate comprehension.

**Figure 5:**
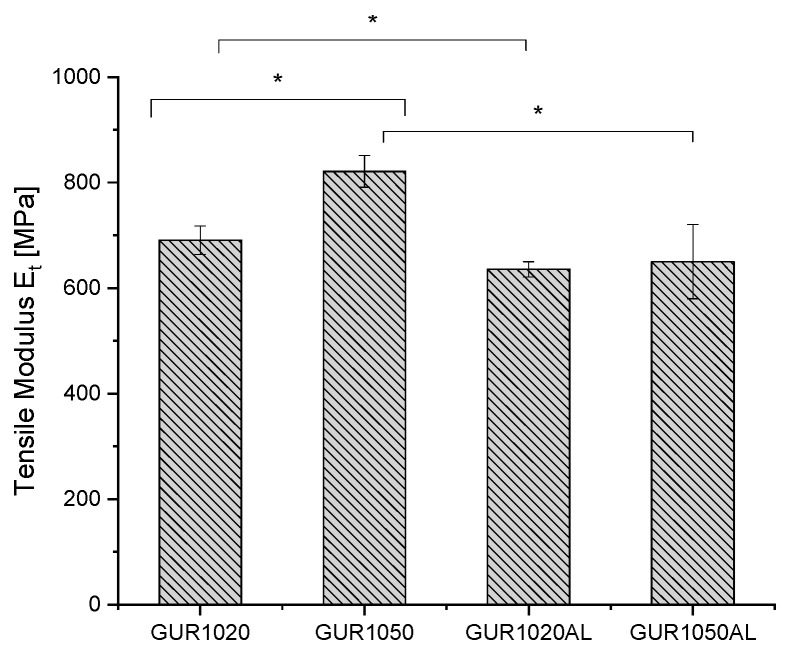
Tensile moduli comparison for various ultra-high molecular weight polyethylene (UHMWPEs), (*) p < 0.05. Measurements taken with a Zwick Z005 universal testing machine in accordance with DIN EN ISO 527, N = 6.

To discover the influence of admixing alendronate on composite stability of the various UHMWPEs, tensile tests were carried out. The stress–strain curves of the tested specimens are shown in Figure 4. The elongation-at-break data reveal various values depending on the GUR: GUR1050 71-121% and GUR1020 150–174%. The tensile modulus exhibited similar behavior—it delivered values ranging from 771 to 848 MPa for GUR1050, and between 647 and 728 MPa for GUR1020. The fracture stress of all the tested specimens fell within a similar range: 34–37 MPa (see Table 4 and Figure 5).

After adding AL, the tensile modules of GUR1020 decreased to 92.1% and that of GUR1050 to 79.1%. The elongation-at-break after adding AL increased in GUR1020 by 22.9% and in GUR1050 by 50.7%. Fracture stress fell by 6.7% in GUR1020 and rose in GUR1050 by 3.5%. 

#### 3.1.5. DSC

The enthalpy of fusion of the samples was determined by means of DSC (see Table 5) in order to determine the crystallinity. The crystallinity of GUR1020 was 52.47 ± 2.41% and that of GUR1050 was 57.56 ± 2.47%. No significant difference was observed between the two values (p > 0.05). The addition of AL changed the crystallinity of GUR1020 to 56.23 ± 2.06% and that of GUR1050 to 55.20 ± 3.46%; neither change was significant at p > 0.05.

### 3.2. Release Experiments

**Figure 6:**
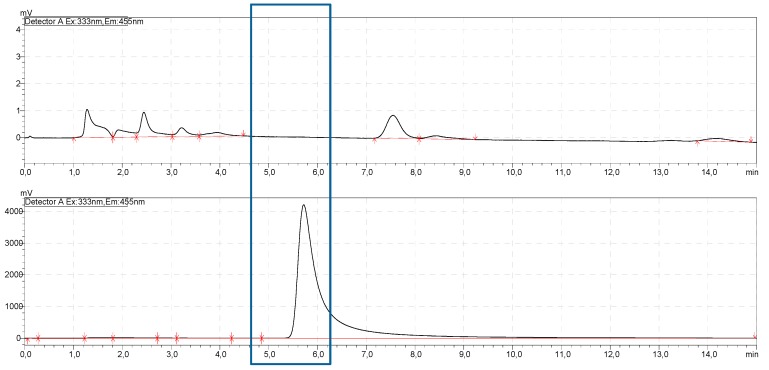
Chromatogram comparison of o-Phtadialdehyde (OPA) (above) and alendronate (AL)–OPA (below). Fluorescence detector: 333 nm excitation and 455 nm emission.

The complex AL–OPA was well determined by HPLC. Compared to the pure OPA peak, the peak of the complex with a peak height over 4000 mV was significantly higher than the pure OPA at 0.1 mV. The complex revealed a retention time of 5.73 ± 0.13 min (see Figure 6) for which there were no peaks in the OPA spectrum. 

The calibration revealed a determination coefficient of 99.63%, which resulted in a detection limit of 0.62 µg/mL and quantification limit of 1.88 µg/mL according to ICH [20].

**Figure 7:**
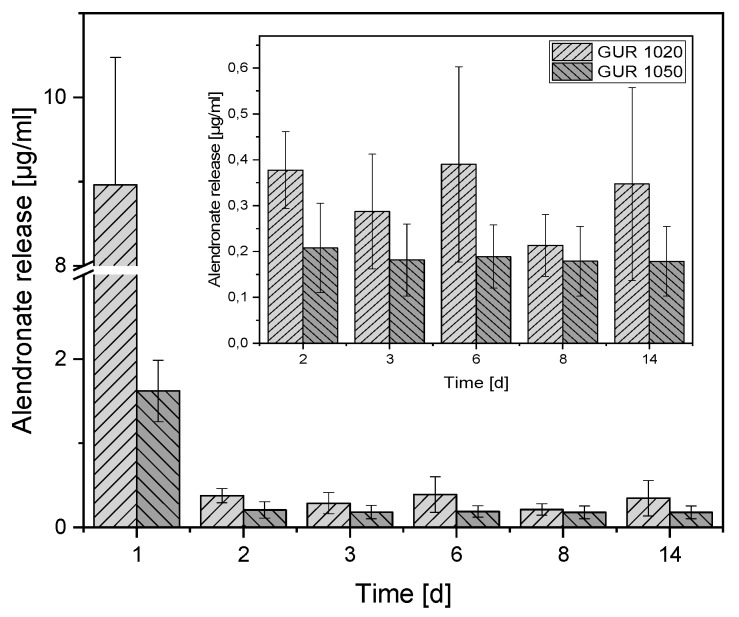
AL release overview; the smaller diagram is an enlargement of our day 2–14 findings to facilitate comparison [N = 30].

The greatest AL release after 24 h is apparent at the beginning: 8.96 ± 1.51 g/mL for GUR1020 and 1.62 ± 0.36 µg/mL for GUR1050 (see Figure 7). After 14 days, 0.34 ± 0.21 µg/mL of AL was released from GUR1020, and 0.17 ± 0.08 µg/mL from GUR1050.

**Figure 8:**
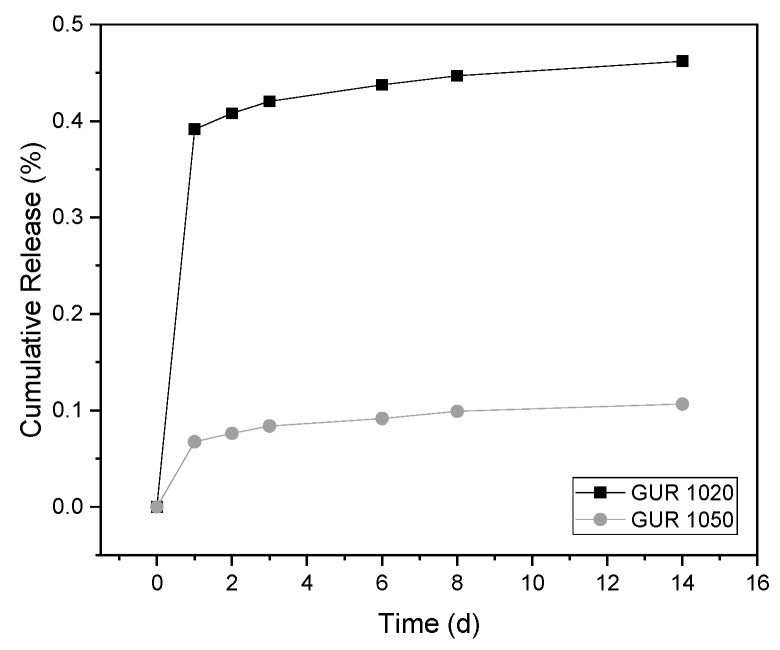
Cumulative AL release depending on the GUR used.

If one considers the cumulative release, it becomes clear that GUR1020 shows a release approx. four times as high as the GUR1050 in relation to the loading quantity. In addition, the release is almost linear from day 3 and has not been completed after 14 days (cf. Figure 8). In both cases, GUR1050‘s 0.1% and GUR1020‘s 0.45% after 14 days indicate a very low proportion of AL was released compared to their initial weight at manufacture. 

### 3.3. Biocompatibility

#### 3.3.1. Live/Dead Assay

**Figure 9:**
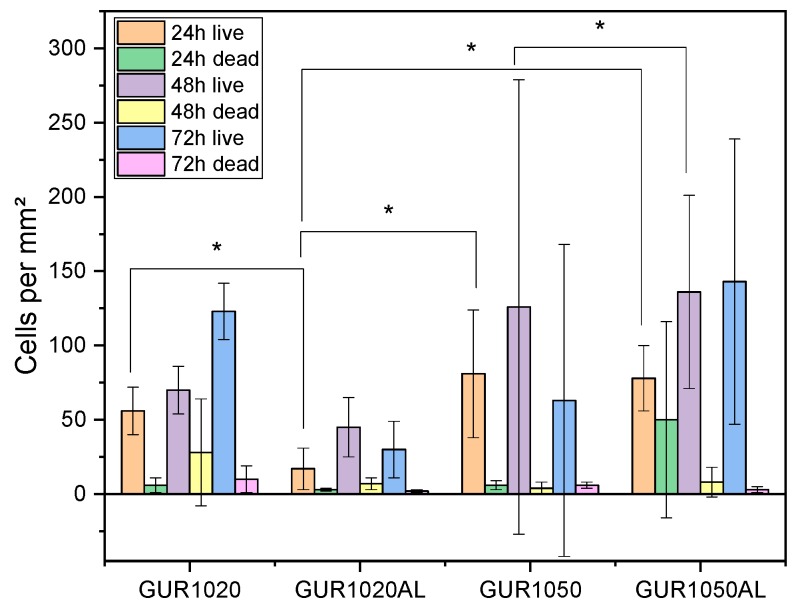
Overview of cells per mm² in conjunction with various materials, *significant difference p < 0.05, N = 15.

The live/dead assay (Table 6 and Figure 9) demonstrated well the increase in cell numbers over time. GUR1050AL exhibited higher cell counts than GUR1020AL. It was also be observed that the cell counts of GUR1020AL were significantly lower than those of pure GUR1020. On the other hand, GUR1050AL did not display such a strong reduction in cell counts compared to GUR1050.

#### 3.3.2. Cell Proliferation (WST)

**Figure 10:**
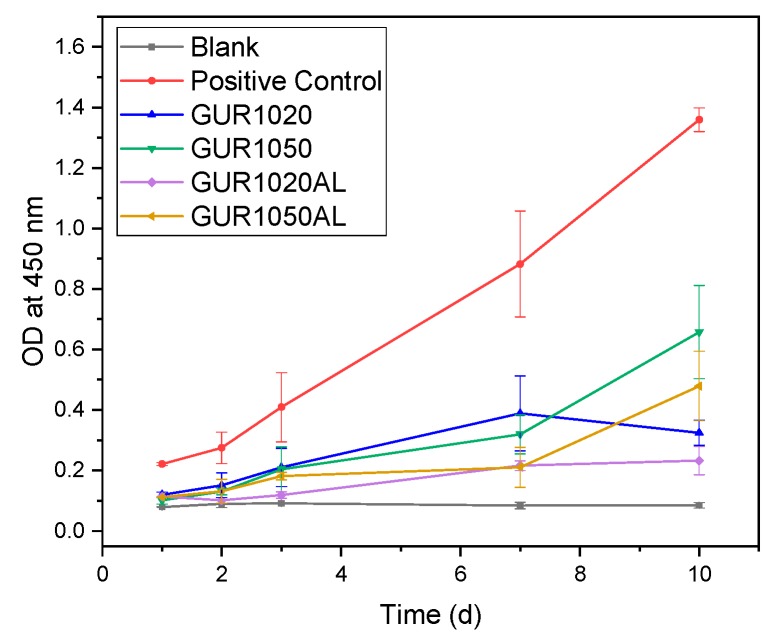
WST test overview, N = 15.

The WST test revealed after 10 days that the cells on all specimens proliferated. Proliferation was, however, not as high as that on Thermanox^TM^ membranes (Nunc, Rochester, USA), which served as our controls. Nevertheless, an increase in proliferation over time was documented. The increases that GUR1050 and GUR1050 demonstrated were highest compared to GUR1020 (see Figure 10).

#### 3.3.3. LDH

**Figure 11:**
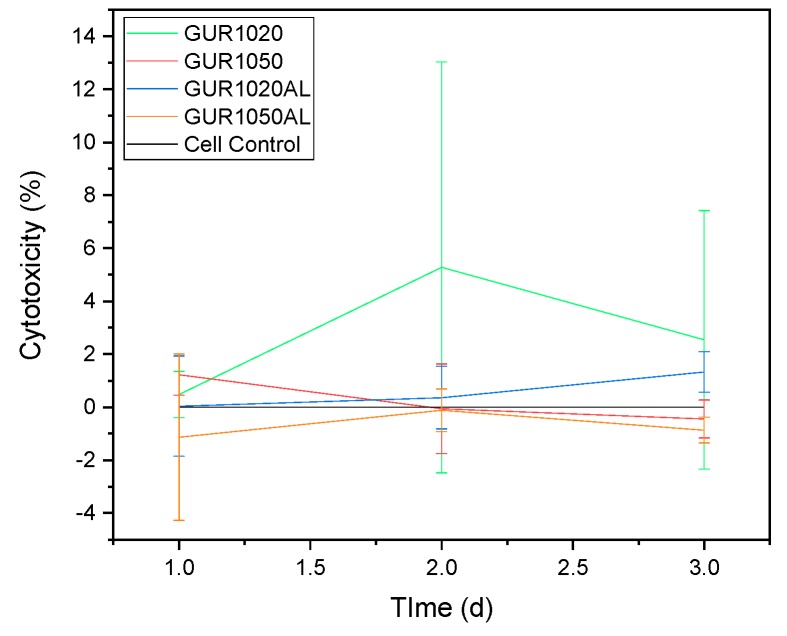
LDH test overview normalized to cell control, N = 15.

LDH revealed no cytotoxicity in the specimens (Figure 11); all values fell within the negative-control range.

## 4. Discussion

### 4.1. Characterization of the Different GUR Types and the Molds

#### 4.1.1. Particle Size Distribution

The GUR particles used by Kumar et al. [21] had a mean particle size of 90 ± 30 µm. Greer et al. [22] unfortunately did not provide any information on the particle size of the GUR used. Gong et al. [15] used a GUR with a mean particle size of 150 µm. The particles we used had a much wider size range. The average particle size for GUR1020 was 54.3 ± 52.5 µm and for GUR1050 was 36.9 ± 38.3 µm. Compared to these values [15,21,22], the GUR types we used for this project showed a significantly smaller particle size.

#### 4.1.2. Surface Roughness

The surface roughness of specimens can affect cell growth [23]. We found no significant difference in surface roughness (S_a_) among the specimens. It is the manufacturing process, which was identical in all our samples, that exerts the strongest influence on surface roughness. A change in this value due to the addition of alendronate can therefore be ruled out.

#### 4.1.3. Tensile Testing

We noted a significant difference in elongation-at-break, in the tensile modules, and in fracture stress among the various GUR types. The findings of Greer at al. [22] were similar. However, they investigated the influence of radiation on crosslinking in UHMWPE. Their elongation-at-break values from native unirradiated specimens were at 396 ± 20% for GUR1020 and 376 ± 52% for GUR1050, much higher than the values we measured. This may be attributable to the difference in manufacturing processes—in this paper, the pressing of UHMWPE+AL having been performed at 140 °C rather than extrusion like Greer et al. [22]. An influence of AL on the mechanical parameters was documented: GUR1020’s tensile module and fracture stress decreased due to the addition of AL. With GUR1050, adding 0.5 wt% AL revealed that the tensile module was also reduced, but fracture stress increased. This factor can be attributed to the reduction in crystallinity. James et al. [17] report a similar relationship between UHMWPE and hyaluronic acid. Crystallinity fell from 52.79 ± 0.08% through the addition of hyaluronic acid to 47.14 ± 0.04%. Comparable values for the tensile moduli for alendronate-containing UHMWPE were also determined by Gong et al. [15], which have dealt with the mechanical and tribological behavior of UHMWPE + AL after mechanical loading. They, however, did not draw comparisons with unloaded UHMWPE. The addition of 0.5 wt% alendronate increased the elongation at break for GUR1020 by 23% and for GUR1050 by 49%. This could be explained by a decreasing crystallinity. 

#### 4.1.4. DSC

The DSC investigations revealed no significant change in crystallinity. There was, however, a slight difference that could have had an influence on release and mechanical characteristics. In the findings of James et al. [17], who investigated changes in UHMWPE crystallinity, the latter was reduced by the addition of hyaluronic acid, but they do not mention whether those changes were significant. We observed that the crystallinity of GUR1020 and GUR1050 lies within the same range of values as reported by Hofmann et al. [24], who investigated the crystallinity of various UHMWPEs. They reported UHMWPE crystallinity values of 2.7 × 10^6^ g/mol of 60% and for UHMWPE with 1.5 × 10^6^ g/mol of 62%. Our crystallinity values lie slightly lower: GUR1020 52.5% and GUR1050 57.6%; however, the UHMWPEs used for this work had higher molar masses: GUR1020 3.5 × 10^6^ g/mol, and GUR1050 5.5 × 10^6^ g/mol

### 4.2. Release Experiments

The alendronate can only be found on the surfaces of the various GUR particles, which are then pressed further in the course of the process. Depending on the mean diameter (d_1020_ = 54.3 ± 52.5 µm; d_1050_ = 36.9 ± 38.3 µm) of the particles, the surface area of the GUR1020 particles (a_1020_ = 9263 µm³) is almost twice as large as that of the GUR1050 particles (a_1050_ = 4278 µm³). The particle size distribution shows a significantly higher value for all other size classes with the GUR1020, except for the very small particles of 0.1–10 µm which predominate with GUR1050 (see Table 1). This effectively increases the surface area of the particles and thus the AL loading, which can explain the much higher AL release from GUR1020. Considering that there is no diffusion in the GUR, except the marginal areas, one comes to the same conclusion. Oral et al. [25] showed similar results, where the question was reversed and vitamin E should diffuse into the GUR. But they also show that a 1-mm deep penetration without special measures can be assumed. The same should then apply to the release as well. This would also explain the relatively minor amount of released alendronate. In contrast to Manoj Kumar et al. [21], the active ingredient was added to the GUR before the moldings were pressed and not impregnated on the surface after the moldings had been fabricated, and they applied gentamycin rather than AL. Their impregnation procedure as well as the different substances used are the reasons for the very pronounced release behavior differences: our 0.5% as opposed to 90% by Manoj Kumar et al. [21]). Qu et al. [16] describe a similar method to produce UHMWPE but at a much higher temperature. Moreover, both Qu et. al [16] and Liu et al. [14] reported wear particles from molds made of UHMWPE with AL that displayed significantly larger surface area than our molds. The larger surface of the wear particles led to a much greater release of AL [14,16]. However, it became clear that the release depends very much on the particle size of the UHMWPE used: GUR1020 released four times as much AL as did GUR1050 despite the fact that both specimens had undergone the same production method and were subject to the same release conditions. A comparative description of the release behavior of different UHMWPE as a function of particle size has not yet been described in the literature.

### 4.3. Biocompatibility

#### 4.3.1. Live/Dead Assay

In the live/dead assay, GUR1050 exhibited much higher cell counts than GUR1020. This is because GUR1020 exhibited greater amounts of AL release and, in line with Im et al. [26], concentrations exceeding 10^−4^ M, leading to a decrease in cell proliferation. However, it was also the case that with decreasing released AL concentrations, an increase in the cell count was observed, similar to what has already been described by Guiliani et al. [27] and Im et al. [26]. Guiliani et al. [27] reported on AL concentrations ranging from 10^−8^ to 10^−13^ mol/L and of etidronate concentrations between 10^−7^ and 10^−9^ mol/L. Im et al. [26] described AL concentrations in the 10^−7^ to 10^−12^ range; the lowest concentration’s effect on proliferation was rather weak. These results were also observed in all live/dead experiments.

#### 4.3.2. WST

In their investigations, Qu et al. [16] und Liu et. al [14] tested the biocompatibility of AL-loaded UHMWPE by using the MTT test instead of the WST test. Their results resembled ours. We observed, exactly as Xiong et al. [28] did, that cell counts rose in relation to AL concentration. In addition, a slight inhibition of proliferation was observed at the beginning, as already described by Reinholz et al. [29], who dealt with the regulation of cell proliferation, differentiation and gene expression of human osteoblasts by bisphosphonates. However, they employed the bisphosphonates etidronate, pamidronate, and zolendronate in their investigations.

#### 4.3.3. LDH

The results from the cytotoxicity assay very slightly exceeded those from our negative controls (cells only). Compared to the wear particle findings of Liu et al. [14], our results (with values of 2% for cytoxicity) also lie within the same range as the controls. Liu et al. studied the in vitro release and cellular response of AL-loaded UHMWPE wear particles. Their controls revealed a 2% cytotoxicity value and a range of 5 to 7% for wear particles containing AL. Thus, our values were clearly below their values for the controls. It was thus possible to prove that no cell toxicity was present at all.

## 5. Conclusions

In the present work, it has been proved that the selection of the UHMWPE has an influence on the release of AL. The particle size of the GUR in particular has a strong influence on release behavior. The way in which the UHMWPE/AL blends are produced, e.g., by pressing instead of extrusion, or the temperature control during pressing at 140 °C instead of 180 °C also influences the release of AL. In addition, an influence of the concentration of alendronate on cell proliferation, as described in the literature [26,27,28], was demonstrated. An AL concentration over 10^4^ M resulted in inhibited proliferation, whereas a falling AL concentration correlated with a rise in cell counts.

## Figures and Tables

**Table 1:** Particle size distribution of the different GUR types [N = 3000].

Particle Size Classes [µm]	Percentage [%]	
	GUR1020	GUR1050
0.1–10	36.52	56.76
11–30	32.61	26.45
31–50	6.78	4.66
51–70	8.06	3.25
71–90	5.65	1.13
91–110	2.41	1.55
111–130	2.33	1.45
131–150	1.81	1.37
151–170	1.95	1.55
171–190	0.82	1.06
191–210	0.68	0.37
211–230	0.15	0.25
231–250	0.15	0.06
251–270	0.08	0
271–290	0	0.06

**Table 2:** Dimensions and Weights of Specimens for Release Testing [N = 10].

Specimen	Width [mm]	Length [mm]	Height [mm]	Weight [g]
**GUR1020**	9.57 ± 0.28	12.72 ± 0.03	2.12 ± 0.02	0.25 ± 0.01
**GUR1050**	9.49 ± 0.31	12.71 ± 0.02	2.11 ± 0.01	0.24 ± 0.01
**GUR1020AL**	9.52 ± 0.20	13.02 ± 0.02	2.01 ± 0.02	0.24 ± 0.01
**GUR1050AL**	9.44 ± 0.42	12.75 ± 0.04	2.13 ± 0.01	0.24 ± 0.01

**Table 3:** Overview of the arithmetical mean height (S_a_) of specimens (N = 10).

Specimens	GUR1020	GUR1050	GUR1020AL	GUR1050AL
**S_a_ [µm]**	2.02 ± 0.43	2.26 ± 0.38	2.57 ± 0.48	2.18 ± 0.27

**Table 4:** Tensile test results (N = 6).

Specimen	E_t_ [MPa]	εt_B_ [%]	σ_B_ [MPa]
**GUR1050**	821 ± 30	71 ± 5	36 ± 10
**GUR1050AL**	650 ± 70	122 ± 116	38 ± 2
**GUR1020**	691 ± 27	151 ± 3	37 ± 1
**GUR1020AL**	635 ± 15	174 ± 7	34 ± 1

E_t_—Tensile modulus; εt_B_—nominal elongation at break; σ_B_—fracture stress.

**Table 5:** Crystallinity percents measured by DSC; N = 4.

Sample	% Crystallinity
**GUR1020**	52.5 ± 2.4
**GUR1020AL**	56.2 ± 2.1
**GUR1050**	57.6 ± 2.5
**GUR1050AL**	55.2 ± 3.5

**Table 6:** Overview of GUR specimen cell counts [N=10].

	Time	cells per mm²					
		24 h		48 h		72 h	
Specimen		live	dead	live	dead	live	dead
**GUR1020**		56 ± 16	6 ± 5	70 ± 16	28 ± 36	123 ± 19	10 ± 9
**GUR1020AL**		17 ± 14	3 ± 1	45 ± 20	7 ± 4	30 ± 19	2 ± 1
**GUR1050**		81 ± 43	6 ± 3	126 ± 153	4 ± 4	63 ± 105	6 ± 2
**GUT1050AL**		78 ± 22	50 ± 66	136 ± 65	8 ± 10	143 ± 96	3 ± 2
